# Talar OsteoPeriostic grafting from the Iliac Crest (TOPIC) for large medial talar osteochondral defects: Operative technique

**DOI:** 10.1007/s00064-020-00673-9

**Published:** 2020-09-09

**Authors:** G. M. M. J. Kerkhoffs, J. N. Altink, S. A. S. Stufkens, J. Dahmen

**Affiliations:** 1 Amsterdam UMC, Location AMC, University of Amsterdam, Department of Orthopaedic Surgery, Amsterdam Movement Sciences, Amsterdam, The Netherlands; 2 Academic Center for Evidence-based Sports Medicine (ACES), Amsterdam, The Netherlands; 3 Amsterdam Collaboration for Health and Safety in Sports (ACHSS), AMC/VUmc International Olympic Committee (IOC) Research Center, Amsterdam, The Netherlands

**Keywords:** Ankle, Cartilage regeneration, Osteochondral autograft, Iliac crest, Transplantation technique, Talus, Knorpelregeneration, Knorpeltransplantat, Beckenkamm, Transplantationstechnik

## Abstract

**Objective:**

Provision of a natural scaffold, good quality cells, and growth factors in order to facilitate the replacement of the complete osteochondral unit with matching talar curvature for large medial primary and secondary osteochondral defects of the talus.

**Indications:**

Symptomatic primary and secondary medial osteochondral defects of the talus not responding to conservative treatment; anterior–posterior or medial–lateral diameter >10 mm on computed tomography (CT); closed distal tibial physis in young patients.

**Contraindications:**

Tibiotalar osteoarthritis grade III; multiple osteochondral defects on the medial, central, and lateral talar dome; malignancy; active infectious ankle joint pathology.

**Surgical technique:**

A medial distal tibial osteotomy is performed, after which the osteochondral defect is excised in toto from the talar dome. The recipient site is microdrilled in order to disrupt subchondral bone vessels. Then, the autograft is harvested from the ipsilateral iliac crest with an oscillating saw, after which the graft is adjusted to an exact fitting shape to match the extracted osteochondral defect and the talar morphology as well as curvature. The graft is implanted with a press-fit technique after which the osteotomy is reduced with two 3.5 mm lag screws and the incision layers are closed. In cases of a large osteotomy, an additional third tubular buttress plate is added, or a third screw at the apex of the osteotomy.

**Postoperative management:**

Non-weight bearing cast for 6 weeks, followed by another 6 weeks with a walking boot. After 12 weeks, a CT scan is performed to assess consolidation of the osteotomy and the inserted autograft. The patient is referred to a physiotherapist.

**Results:**

Ten cases underwent the TOPIC procedure, and at 1 year follow-up all clinical scores improved. Radiological outcomes showed consolidation of all osteotomies and all inserted grafts showed consolidation. Complications included one spina iliaca anterior avulsion and one hypaesthesia of the saphenous nerve; in two patients the fixation screws of the medial malleolar osteotomy were removed.

## Abstract (de)

**Operationsziel:**

Wiederherstellung des natürlichen Knochengerüsts mit gesunden Zellen und Wachstumsfaktoren zur Rekonstruktion von großen primären und sekundären Knochendefekten an der medialen Talusrolle, mit Einsatz einer kompletten osteochondralen Einheit mit passendem Radius entsprechend der Talusrolle.

**Indikationen:**

Symptomatische primäre und sekundäre mediale osteochondrale Defekte am Talus nach fehlgeschlagener Physiotherapie; anteroposteriorer und/oder mediolateraler Durchmesser >10 mm in der Computertomographie (CT); bei jüngeren Patienten: geschlossene Wachstumsfuge der distalen Tibia.

**Kontraindikationen:**

Generalisierte Osteoarthrose im gesamten Sprunggelenk; multiple Osteochondraldefekte; aktive Infektion; Malignität.

**Operationstechnik:**

Zunächst wird eine mediale Osteotomie der distalen Tibia durchgeführt, anschließend der Osteochondrale Defekt vollständig aus dem Talus entfernt. Der Defekt wird angebohrt, um die subchondralen Blutgefäße zu öffnen. Das Transplantat wird am ipsilateralen Beckenkamm mit der oszillierenden Säge entnommen und in den Defekt unter Beachtung der korrekten Wiederherstellung von Form und Radius der Talusrolle eingepasst. Dann wird das Transplantat mittels Press-Fit-Technik fixiert. Anschließend erfolgen die Reposition der Tibiaosteotomie und die Fixation mit zwei 3,5-mm-Zugschrauben sowie der Wundverschluss.

**Weiterbehandlung:**

Unbelastete Mobilisation im Unterschenkelgips für 6 Wochen, danach Vollbelastung in einer Unterschenkelorthese (Walker) für weitere 6 Wochen. Nach 12 Wochen Kontroll-CT zur Analyse der Knochenheilung sowohl der medialen Tibiaosteotomie als auch der Integration des Transplantates in den Talus. Danach beginnen mit Physiotherapie.

**Ergebnisse:**

In einer prospektiven Studie wurden 10 konsekutive Patienten mit der TOPIC-Technik behandelt. Nach einem Nachuntersuchungszeitraum von 12 Monaten verbesserten sich alle klinischen Werte der Patienten. Als Komplikationen sind bisher eine Avulsionsfraktur der Spina iliaca anterior und eine Hypästhesie des N. Saphenus aufgetreten. Bei 2 Patienten traten leichte Druckschmerzen über dem medialen Malleolus auf, die nach Entfernen der Zugschrauben wieder verschwanden.

## Introductory remarks

Osteochondral defects of the talus are defined as damage to the talar articular cartilage and its subchondral bone. The origin of this injury may be due to ankle fractures and ankle sprains, an avascular episode, and possibly a genetic predisposition [1, 15, 25, 50]. The injuries have a severe impact on the quality of life of active patients, due to deep ankle pain during weight-bearing and sporting activities [4, 5, 22].

Primary management of these defects is conservative; surgery is considered in cases of persistent symptoms [52]. For small primary defects, common first-line surgical management options consist of (arthroscopic) bone marrow stimulation and retrograde drilling [11–13, 30, 43]. For fixable defects, arthroscopic and open internal fixation procedures are amenable options and have proven to be effective for pediatric and adult patient populations [28, 29, 31, 45, 46]. In case of large defects or in case of failure of first-line surgical treatment, more extensive and invasive surgery can be considered a necessary step in the management process [30]. The more common treatment strategies currently include osteochondral allograft procedures, osteochondral autograft transfer system (OATS) procedures, and classic and matrix-associated chondrocyte implantation (ACI, MACI) [20, 21, 32, 39, 55, 56]. Even though the OATS procedure is regarded as an effective surgical management option with a 90% success rate reported in the literature, donor-site morbidity has been reported to occur relatively frequently with incidence rates ranging from 11 to 35% when the graft was harvested from the ipsilateral femoral condyle [13, 16, 19, 23, 24, 26, 44, 57]. As an alternative to an OATS procedure harvesting the graft from the knee, an autologous osteoperiosteal cylinder graft can be harvested from the iliac crest. This technique has been described by Hu et al. [26] and Chen et al. [10]. This surgical treatment yielded highly promising results with good clinical follow-up scores and radiological outcomes. However, both author groups utilized cylindrical grafts, thereby compromising optimal individualized treatment for large talar osteochondral defects as it is known that that the size of the cylinder autograft is static and determined preoperatively.

In order to overcome the aforementioned disadvantages, we developed a new surgical technique for the treatment of large talar osteochondral defects or secondary lesions: the Talar OsteoPeriostic grafting from the Iliac Crest (TOPIC) procedure. This technique utilizes the ipsilateral iliac crest with its overlying periosteal layer as a harvesting location for the autograft. The use of the periosteum has been shown to have potential concerning articular cartilage-like tissue regeneration [35, 40]. The chondrogenic potential of this technique arises because the cambium layer of periosteum contains chondrocyte precursor cells [27, 37]. A bone-periosteal transplant for bone–cartilage repair meets the three requirements for tissue engineering: a source of cells, a scaffold, and local growth factors [2, 18, 34, 47, 54]. In addition, the curvature of the iliac crest is highly similar to the curvature of the talar bone, and the harvested graft can be exactly fitted to the excised defect from the talus [38]. The goal of the present surgical technique description is to describe the surgical technique of the newly developed TOPIC procedure and evaluate potential pearls and pitfalls.

## Surgical principle and objective

A large primary or secondary osteochondral defect to the talus is a challenging problem in the orthopedic clinic. Multiple techniques exist to treat these defects, ranging from a cartilage implantation technique to an OATS procedure as well as an allograft procedure. These techniques have their individual advantages and disadvantages—such as donor-site morbidity, poor quality subchondral bone repair and low clinical efficacy. The current paper describes a new surgical technique for the treatment of large primary and secondary medial talar osteochondral defects. The technique comprises a medial distal tibial osteotomy and a talar autograft transplantation from the ipsilateral iliac crest. The technique has the main advantage of providing a natural scaffold, good quality cells, and growth factors, facilitating the replacement of the complete osteochondral unit—a triad considered important in the treatment of these defects.

## Advantages

- No expensive planning software required
- Single-stage technique
- Limited operation time
- Cost-effective
- Surface geometry talus and curvature crest [38]
- Iliac crest graft provides a scaffold, cells, and growth factors [2, 18, 34, 47, 54].

## Disadvantages

- Access to talar dome through an osteotomy
- Potential complaints of discomfort of the inserted screws and/or plate
- Complications specifically for the harvest site, the iliac crest, and hyp(er)aesthesia of the skin [14]

## Indications

- General: painful osteochondral defect with a clear episode of deep ankle pain not responding to conservative treatment
- Anterior–posterior or medial–lateral diameter should exceed 10 mm on computed tomography (CT)
- The depth of the defect is no limitation for this procedure
- Both primary and secondary surgical lesions are treated with a TOPIC
- Closed distal tibial physis in young patients
- If concomitant instability is present, a stabilizing procedure will be considered
- In case of malalignment, a corrective osteotomy will be considered.

## Contraindications

- Tibiotalar osteoarthritis grade III
- Multiple osteochondral defects on the medial, central, and lateral talar dome
- Active infectious ankle joint pathology
- Malignancy

## Patient information

- Usual surgical risks include infection, hematoma, thromboembolic event, wound healing problems
- Transient or permanent nerve damage leading to hypaesthesia of the saphenous nerve
- Late or early screw and or plate discomfort requiring removal after consolidation
- Nonweight bearing cast for 6 weeks, followed by a walking boot for another 6 weeks

## Preoperative work up

The Department of Orthopedic Surgery of the Amsterdam UMC, Location AMC has been officially recognized as an expert center for the treatment of osteochondral defects of the ankle and foot. All patients are screened with a careful patient history and physical examination of both lower legs. During the physical examination there is special attention for recognizable pain on palpation over the site of the talar osteochondral defect with the foot in plantar flexion to be sure that the patients’ deep ankle pain indeed arises from the osteochondral defect and not from any other pathology. Special emphasis is also put on the range of motion of the ankle joint, especially plantar flexion. This is done in order to determine whether an osteotomy will be necessary or whether the total defect can be reached without an osteotomy.

For confirmation of the diagnosis and as a preoperative sizing and planning tool, an additional CT scan is made. All talar osteochondral lesions are scanned for defect size, location, shape, and morphology. In addition, the lesion’s preoperative shape is classified according to the modified Berndt and Harty’s staging system with added stage V by Scranton and McDermott [7, 51].

## Instruments and implants

- Hohman retractors
- Oscillating saw and/or chisel with thin blades
- Beaver knife
- 2.0 mm Kirschner wires
- 2.0 mm drill
- Coagulation knife
- Hemostatic gelatin sponge (Spongostan®)
- Impactor
- 3.5 mm cortical screws or a headless alternative
- Standard orthopedic set
- Chisel set
- Large Weber clamps

## Anesthesia and positioning

- General or spinal anesthesia
- Supine position with a tourniquet applied around the thigh
- Antibiotic prophylaxis (cefazolin 2 g intravenous injection) is administered to each patient

## Surgical technique

**Fig. 1:**
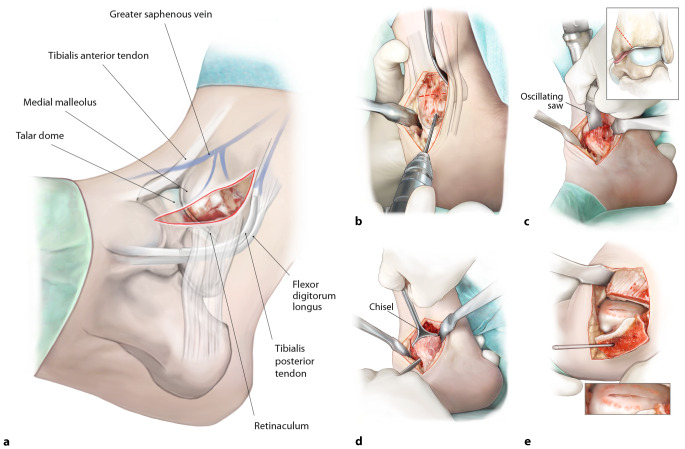
*Medial distal tibial osteotomy*. After prepping and draping, a slightly curved incision of approximately 7 cm is made over the medial malleolus (**a**), the large saphenous vein is identified and protected. An anteromedial arthrotomy is performed with a partial resection of the anteromedial joint capsule. Posterior from the medial malleolus, the retinaculum of the posterior tibial tendon is opened and the tendon is retracted to allow a subtle posteromedial capsular resection. Anterior and posterior Hohman retractors are placed in order to protect the neurovascular structures and the tendons. Then, an osteotomy is first prepared by predrilling the lag screw holes (**b**). Thereafter, the osteotomy is performed with an oscillating saw (**c**). In order to prevent thermal damage to the cartilage, the last 3 mm of the osteochondral zone of the distal tibial plafond is finished with a chisel (**d**). The extent of the osteotomy is decided based on the exact localization of the defect, as well as the sagittal and the coronal diameter of the defect. After finishing the osteotomy, the osteotomized distal tibia part is dislocated in both medial and plantar direction and this position is secured with the use of 1 (or 2) 2.0 mm Kirschner wire(s). This facilitates full exposure of the medial to central talar dome (**e**)

**Fig. 2:**
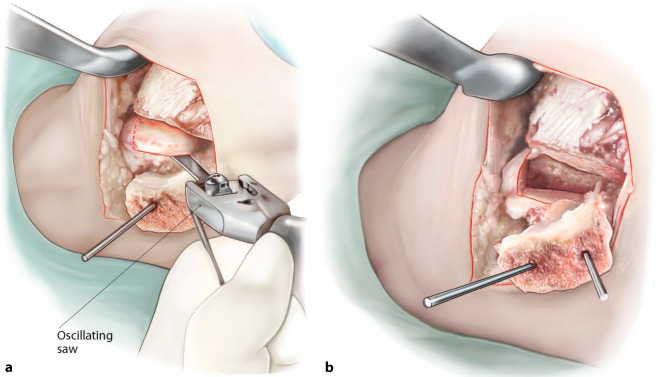
*Excision of the diseased osteochondral defect.* After the identification and exposure of the defect, a rectangular incision of the cartilage is made to preserve as much healthy cartilage as possible and to prevent the cartilage from rupturing in the next steps of the procedure (**a**). All cystic and necrotic bone is removed in total with the use of either an oscillating saw or a chisel with thin blades or both. The end result should be a total removal of the premeasured talar diseased osteochondral defect, deep enough so that healthy bone can be observed (**b**)

**Fig. 3:**
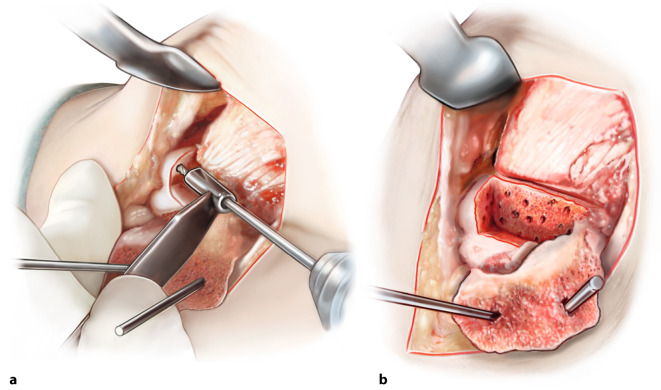
*Drilling of the recipient’s subchondral bone base*. As the defect has been excised beyond the subchondral bone plate, the base of the autograft recipient can be freely (micro)drilled for bone marrow stimulation using a 2.0 mm drill (**a**). This step of the procedure disrupts intraosseous vessels so that blood and bone marrow cells are introduced into the yet empty defect. **b** End result after drilling of the subchondral bone

**Fig. 4:**
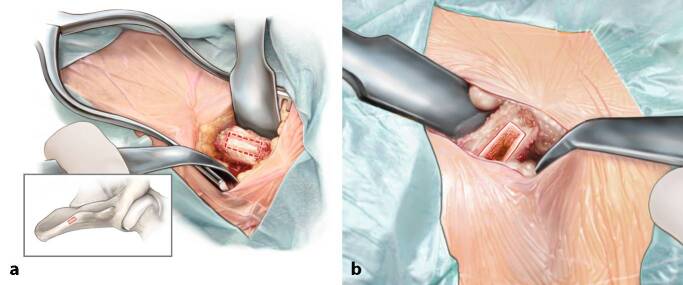
*Measuring and harvesting the donor autograft from the iliac crest.* After having drilled the subchondral bone of the talus, the graft harvest is started. The first step is to exactly measure the size of the excised block, so that a correct approximation of the donor autograft can be performed—the graft should be 1 mm larger in all directions (anteroposterior and mediolateral diameter as well as depth). Approach the iliac crest through a horizontal incision of approximately 3.5 cm. Take care not to use the coagulation knife in order to keep the periosteum nicely intact and attached. Expose part of the iliac crest by means of two retractors (**a**). Then, a monocortical, bicortical, or tricortical osteoperiosteal autograft from the ipsilateral iliac crest is harvested with an oscillating saw and chisel (**b**). The combination of the size of the graft needed and the width of the crest dictates the choice for monocortical, bicortical, or a tricortical graft. A hemostatic gelatin sponge (Spongostan®) can be left in the iliac crest after harvesting the autograft

**Fig. 5:**
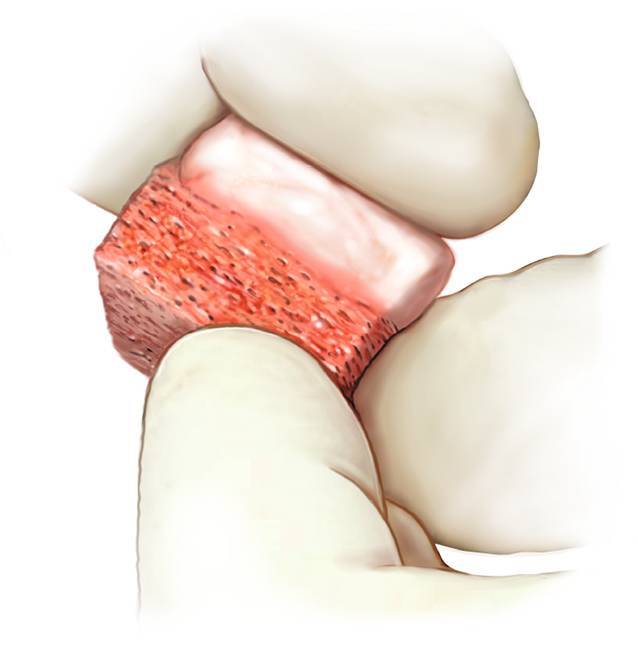
*Adjusting the fitting shape of the autograft.* The graft can be further fine-tuned to optimally fit curvature, size, and depth of the talar defect. Take care that graft is not too proud: 1–2 mm under the talar cartilage level at all sides seems best in order to prevent over-stuffing. The graft should be placed press-fit, which means 1 mm should be added on all dimensions of the excised defect, as mentioned in Fig. 4. Take time to perform this correctly as it will facilitate the step of executing a press-fit insertion technique. Comparing the graft to the excised talar defect may ameliorate the similarity of the autograft

**Fig. 6:**
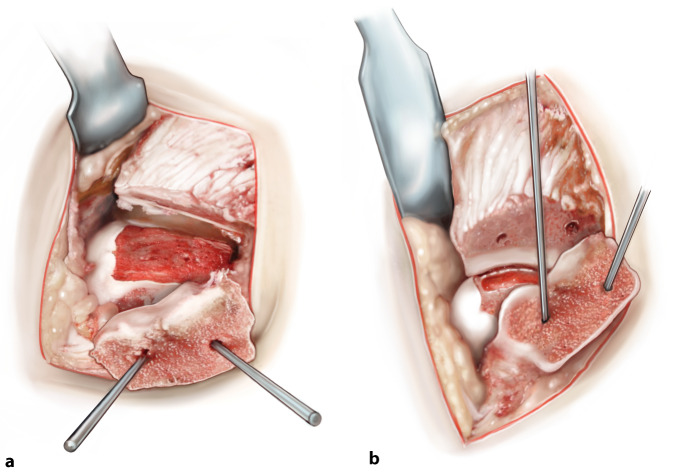
*Implanting the graft through a press-fit technique.* Once the optimal fitting shape has been reached, the autograft is transported and placed into the host site (**a**). By means of an impactor the inserted autograft can be fitted exactly 1–2 mm underneath the level of the talar cartilage. The curvature of the iliac crest accurately matches the talar curvature (**b**). As this is a press-fit technique, no additional screws are necessary in order to fixate the autograft. In case of graft instability, the option of using one or two screws to stabilize the graft always remains

**Fig. 7:**
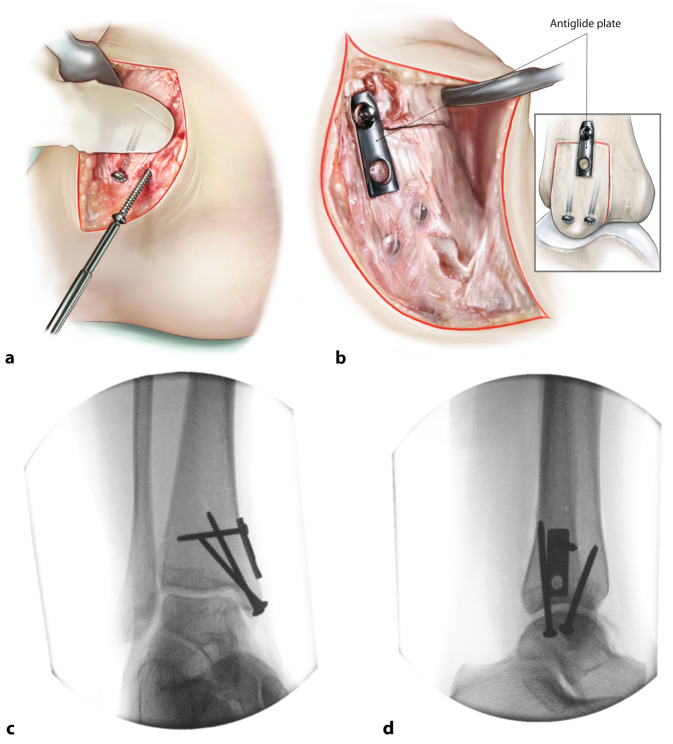
*Closure of the distal tibial osteotomy and incision layers.* The distal tibial osteotomy is closed and fixated by means of two 3.5 mm lag screws in case of a standard small medial malleolar osteotomy. We aim for two divergent bicortical screws (**a**). In case of a larger distal osteotomy we opt for two bicortical medial screws perpendicular to the osteotomy and an antiglide plate to prevent rotation and proximal translation of the osteotomy (**b–d**). Sometimes a third screw at the apex of the osteotomy is also a suitable option. Rinsing and closure in layers follows

(Figs. 1, 2, 3, 4, 5, 6 and 7)

## Special surgical considerations

- A preoperative CT scan is used to measure the bony dimensions of the osteochondral defect (anteroposterior diameter, mediolateral diameter and depth)
- The last part of the distal tibial osteotomy is done with a chisel to prevent thermal damage to the osteochondral unit
- Both the distal tibial osteotomy and iliac crest autograft transplantation can be customized for every patient
- In case a larger distal tibial osteotomy is indicated, an extra antiglide plate can be used to prevent rotation and proximal translation
- Press-fit autograft transplantation allows for quick rehabilitation without need for intra-articular screw removal
- Theoretically, an optimal graft to replace an osteochondral unit has the following characteristics: natural scaffold, good quality cells, and growth factors [36, 41, 42, 48, 53]

## Postoperative management

Directly after surgery a lower leg splint is provided for the first 24 h. Surgery is performed as a 1-day admission as this allows for adequate pain management and application of a fresh circular nonweight bearing lower leg cast applied for 2 weeks. After 2 weeks, the stitches are removed and the patient is allowed an hour of dorsoplantar ankle flexion motion in order to prevent stiffness and possibly enhance the stimulation of progenitor cells from the periosteum to produce an optimal chondral layer following stimulation through ankle joint motion [8, 9, 33].

The non-weight bearing period is 6 weeks with regular cast changes in order to allow an hour of ankle joint motion. At the 6‑week follow-up, a walking boot is applied for another 6 weeks. At the 12-week follow-up, a computed tomography (CT) scan provides information on consolidation of the distal tibial osteotomy as well as the autograft. At this visit we also do a clinical assessment of the patient and provide an individualized guideline for the next 3 months of rehabilitation. The patient is referred to a physiotherapist to guide and stimulate clinical progress and aid in the rehabilitation process. Additional follow-ups are performed at 6 months and 1 year postoperatively in order to closely assess progress of the patient, also concerning return to work, sports, and performance.

## Errors, hazards, complications

At every follow-up, patients are checked for potential complications, such as crest pain, hyp(er)aesthesia in the crest and ankle joint region, infections, ankle joint synovitis, and postoperative pain. A radiograph is obtained 6 weeks postoperatively to assess consolidation of the osteotomy. A CT scan is made at 3 and 12 months after surgery to assess consolidation, ingrowth, and talar remodeling of the transplant. Potential pitfalls include the following:

- Cartilage damage to ankle joint if not enough attention is paid to the distal tibial osteotomy
- Neurovascular or posterior tibial tendon damage if Hohman retractors are not placed properly
- Avulsion or fracture of the iliac crest when harvesting the graft too close to the anterior superior iliac spine
- Proudness of the autograft might give a kissing lesion or chondral wear to distal anteromedial corner of the ankle joint
- Graft failure
- Non- or malunion of the osteotomy
- Ankle joint stiffness
- Persistent ankle pain because of progressive osteoarthritis (OA)
- Impingement of the medial gutter if the graft has slight overhang or in case of presence of an osteophyte or multiple osteophytes

## Results

**Fig. 8:**
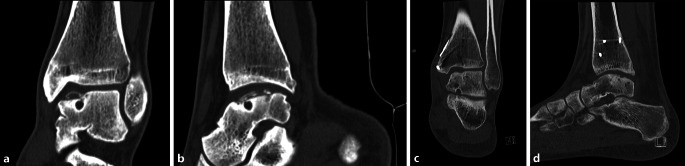
Preoperative and postoperative (1 year follow-up) computed tomography (CT) scans of the same patient. **a** Coronal preoperative CT scan. **b** Sagittal preoperative CT scan. **c** Coronal postoperative CT scan. **d** Sagittal postoperative CT scan

The present study was approved by the local Medical Ethics Committee at the University of Amsterdam and performed in accordance with the current ethical standards (Declaration of Helsinki, reference number MEC 14/237 #14.17.0288). All patients undergoing a TOPIC procedure were assessed preoperatively and at 1 year postoperatively. Ten patients underwent the TOPIC procedure. Mean age was 36.1 years (standard deviation [SD] 17.9). The mean follow-up for the patients was 12 months (SD 1.0). Pre- and postoperative assessment included the Foot and Ankle Outcome Score (FAOS) subcales, the American Orthopaedic Foot and Ankle Society (AOFAS), Short-Form 36 (SF-36) Mental Component Scale (MCS) and Physical Component Scale (PCS), and the Numeric Rating Scales (NRS) of pain at rest and during walking and stair-climbing. Dual energy CT scans were taken preoperatively and at the 1 year follow-up. All patients were available for follow-up. The AOFAS significantly improved from 50 to 83 (*p* = 0.02). The NRS during rest improved from 2.4 to 1.0 (*p* = 0.072), during walking from 4.6 to 2.3 (*p* = 0.009), during stairclimbing from 4.3 to 1.8 (*p* = 0.011), and during running from 7.1 to 2.6 (*p* = 0.0011). The SF-36 PCS improved from 36 to 48 (*p* = 0.001) and the MCS from 47 to 56 (*p* = 0.132). All Foot and Ankle Outcome Score (FAOS) subscales improved significantly, except for the symptoms subscale. On the postoperative CT scans, all osteotomies and grafts showed consolidation (Fig. 8). Complications included one spina iliaca anterior avulsion and one temporary loss of sensitivity of the saphenous nerve; in two patients the medial malleolar screws were removed.

We present a novel press-fit autograft transfer technique for large talar osteochondral defects. Talar OsteoPeriostic grafting from the Iliac Crest (TOPIC) proved to be a reproducible and safe technique, with good short-term functional results and early radiographic consolidation of the graft on CT scan. We consider an improvement of on average of 30 points on the AOFAS scale and 2.3 (during activity) and 4.5 points (during running) on the NRS very promising. This, as a mean improvement of −2.0 on the NRS scale of 0 to 10 is significantly associated with the concept of a “much better improvement” [49].

Further advantages of the technique are its relative ease to perform, limited operation time, and the costs being relatively low in comparison to other extensive techniques such as for example an allograft implantation technique or chondrogenesis-inducing techniques. In addition, the iliac crest as a harvesting site not only matches the talar surface geometry, but it also has a chondrogenic potential to regenerate articular cartilage utilizing periosteum [27, 40]. If a TOPIC does fail, various salvage procedures are available: a redo, an ankle fusion or a total ankle prosthesis.

Disadvantages are the use of an autograft of the ipsilateral iliac crest that has potential complications such as persistent pain, neuralgia, or contour loss of the crest; however, with the technique used for harvesting the graft, this is minimized [3, 14]. Another disadvantage might be the use of a fairly large medial distal tibial osteotomy that has to extend into the tibial plafond in order to be able to excise the entire defect. All medial malleolar osteotomies showed complete union at 3 months postoperatively. Another complication of the present technique may be the complaints of the medial malleolar screws that were inserted [6, 17]. We encountered this in 3 patients, and hardware removal was necessary in 2 patients. A further disadvantage of the TOPIC technique might be the requirement of a high level of surgical skill for exact graft shaping. It needs to be 0.5 mm oversized in the sagittal plane while the width and depth in the coronal and axial plane should exactly fit the depth of the created defect in order to allow the graft to be placed 1–2 mm subchondral after impaction.

In a recent review, we described that there are several options that appear to be safe and provide good results in prospective case series, with up to 90% success of OATS techniques for larger talar osteochondral defects after failed prior surgical treatment [30]. In these OATS results, there were several harvesting sites described for the plugs. The iliac crest as a graft site has been previously described with good results; however, all techniques have the potential disadvantage of a nonfully anatomical fit and the possibility of new cyst formation in medium and long-term follow-up along the plugs used [26].
